# Environmental Risks Perception Among Citizens Living Near Industrial Plants: A Cross-Sectional Study

**DOI:** 10.3390/ijerph17134870

**Published:** 2020-07-06

**Authors:** Marco Dettori, Paola Pittaluga, Giulia Busonera, Carmelo Gugliotta, Antonio Azara, Andrea Piana, Antonella Arghittu, Paolo Castiglia

**Affiliations:** 1 Department of Medical, Surgical and Experimental Sciences, University of Sassari, 07100 Sassari, Italy; gugliotta.melo@gmail.com (C.G.); azara@uniss.it (A.A.); piana@uniss.it (A.P.); castigli@uniss.it (P.C.); 2 Department of Architecture Design and Urban Planning, University of Sassari, 07100 Sassari, Italy; pittaluga@uniss.it (P.P.); giulia.busonera@hotmail.it (G.B.); 3 University Hospital in Sassari, 07100 Sassari, Italy; 4 Department of Biomedical Sciences, University of Sassari, 07100 Sassari, Italy; arghittu.antonella@gmail.com

**Keywords:** risk perception, community outrage, environmental risks perception, environmental health

## Abstract

The present work is a cross-sectional study aimed at assessing the risk perception and evaluating the community outrage linked to environmental factors among a self-selected sample of citizens living in an area characterized by the presence of industrial structures of high emotional impact. An anonymous questionnaire was administered to the population by publishing a Google form URL code in local and regional newspapers and via social media. The resulting data were entered on Excel and analyzed. Qualitative variables were summarized with absolute and relative (percentage) frequencies. The results showed that the event that causes the greatest worry was air pollution, with 92.6% of the respondents stating that they perceived the problem as “very” or “quite” worrying. Furthermore, all the health problems investigated in relation to environmental quality aroused concern among the interviewees, with 93.1% believing there was a cause-effect relationship between environmental quality and health. Overall, as other studies had previously underlined, the survey shows that the perceived risks are not always in line with the real ones, Thus, it is imperative to articulate interventions aimed at offering the population objective tools to enable them to interpret the risks themselves. In this regard, a fundamental role is played by adequate communication between the competent bodies and political decision-makers and the population.

## 1. Introduction

The relationship between environment and health is of extreme relevance in Public Health. According to the World Health Organization (WHO) [1], 23% of all deaths globally are attributable to environmental factors, and several diseases could be avoided if we lived in healthier environments. In particular, according to the European Environmental Agency [2], poor air quality causes 6.5 million premature deaths worldwide, 620,000 of which are in the WHO European Region. Indeed, the European Environment Agency [3] also certifies that noise and air pollution continue to have a serious impact on the health of the population, and human activities (mainly the key sectors of industry, energy, transport, agriculture) are a source of strong environmental pressure [4,5,6,7].

The growing awareness of the health impacts caused by the alteration of environmental conditions by anthropic activities, such as industrial expansion near urban areas, atmospheric pollution, and climate change, plays a key role in the judgment and acceptability of the risks related to environmental quality [7,8,9,10,11]. In particular, the perception of the risk linked to environmental factors can be interpreted as a combination of objective (such as the levels of real exposure to a danger) and subjective factors (i.e., assessments arising from education, culture, values, personal beliefs, and perception of reality) [12]. The social, political, and decision-making dynamics that surround the event also contribute to these factors [13,14,15,16,17,18].

For these reasons, environmental determinants known to have a negative impact on health often cause the real health risk attributable to them to be overestimated [3,4,15]. Social context, convenience, and participation in political choices can influence the perception of an event [19,20,21,22,23,24,25], in the same way as ineffective risk communication and a poorly informed population [17,19,26]. This aspect is well described by the American sociologist Peter Sandman’s “Hazard vs. Outrage” theory, according to which the perception of risk is closely linked to the emotional component surrounding the event (community outrage) [27].

A well-known phenomenon of a purely emotional and perceptual nature is explained by the NIMBY (Not In My Back Yard syndrome), a phrase that first appeared in the 1970s to describe the common opposition to the siting of works that, although of public utility, nobody is willing to accept in the proximity of their living environment [28,29]. Since this phenomenon was identified, numerous studies in the literature have shown that the presence of environmental factors with high emotional impact (e.g., incinerators, waste-to-energy plants, and wind farms) is linked to the community outrage and risk perception [13,14,30].

On the basis of these premises, this work is a cross-sectional study aimed at assessing the risk perception and evaluating the community outrage linked to environmental factors among a self-selected sample of citizens living in an area characterized by the presence of industrial structures of high emotional impact.

## 2. Materials and Methods

The present study did not require ethical approval for its observational design according to Italian law (Gazzetta Ufficiale no. 76 dated 31.3.2008).

### 2.1. Study Area

The study was conducted in Sardinia, an Italian region with a population of 1,648,176 inhabitants (810,072 males; 838,104 females; average age of 45.9 years) as of 2018 [31]. Table 1 shows the distribution of the Sardinian population by age, gender, and marital status [32].

Owing to its insularity, the region lends itself very well to observational investigations and represents an excellent test case in relation to the reported social dynamics. In fact, the island has already proven to be well suited to epidemiological studies as it preserves the region from interferences caused by territorial contiguity. As such, it can act as an excellent exercise for the reported social and epidemiological dynamics.

**Figure 1:**
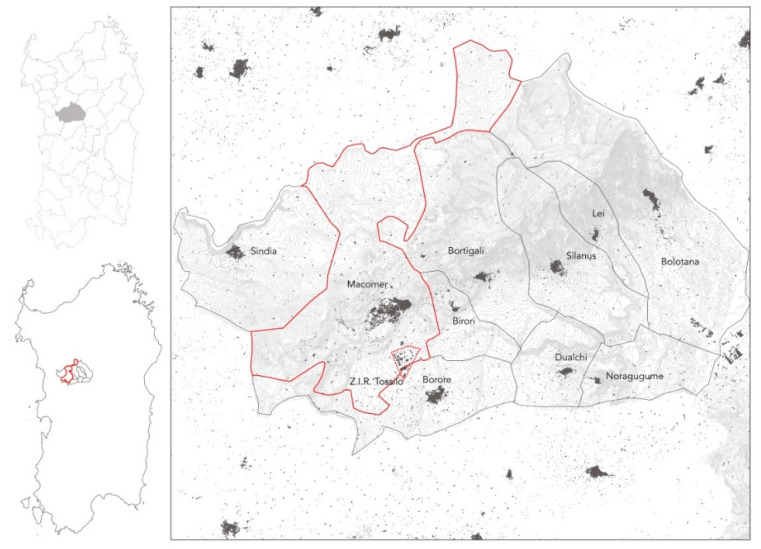
Geographical location of the 10 Municipalities of Marghine, Sardinia, Italy and the population in 2018. In red, the Municipality of Macomer, the most heavily populated in Marghine and the industrial zone (Z.I.R.—Zona Industriale di interesse Regionale) of Tossilo.

In particular, the present study was conducted in the Marghine area, a historical region in central Sardinia which covers an area of 475.42 km^2^ and includes 10 municipalities: Birori, Bolotana, Borore, Bortigali, Dualchi, Lei, Macomer, Noragugume, Silanus, and Sindia [31,32,33,34]. Figure 1 shows the study area’s territorial framework and the population of each municipality in 2018, the year in which observation was carried out, whereas Table 2 reports total inhabitants in Marghine and in the individual municipalities as of 2018 [34].

**Figure 2:**
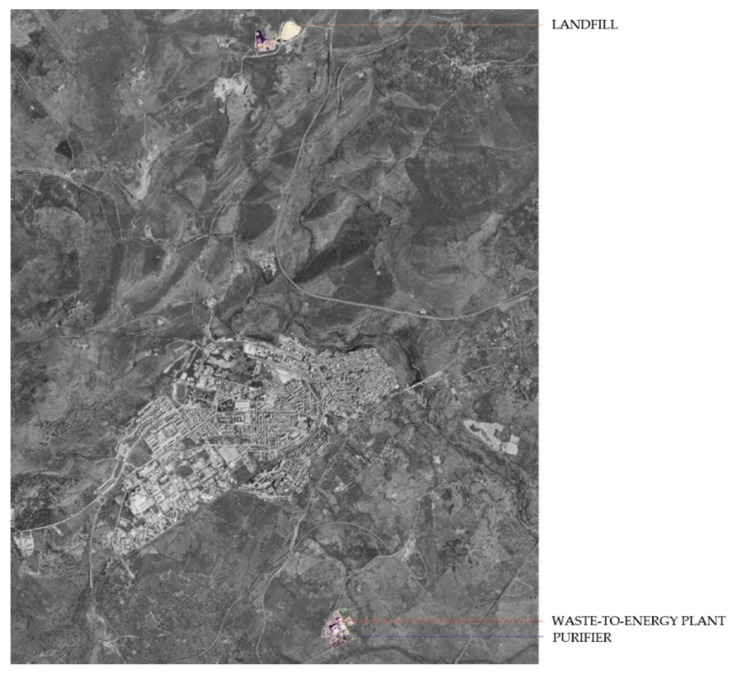
Location of industrial waste management plants near the urban area of Macomer, the most populated municipality in Marghine.

As shown in Figure 2, several industrial plants (mostly dedicated to the management of solid waste from the entire Region) are situated in Marghine, located near urban areas, in particular in the Municipality of Macomer, the main town in the area, with a population of 10,019 in 2018.

Figure 1 and Figure 2 show that Tossilo, an industrial zone (Z.I.R.—Zona Industriale di interesse Regionale), is located within the municipal territory of Macomer. Here there is a waste-to-energy plant, currently under expansion. The new plant will consist of a 30-Megawatt waste-to-energy line, capable of ensuring a treatment capacity of approximately 7.64 tons per hour, corresponding to 183.36 tons per day, with an average calorific value of 13,180 kilojoules per kilogram [35,36].

In the same industrial zone there is also a purifier that treats the waste water from the waste-to-energy process [36,37,38]. Finally, the production cycle also includes the management of the controlled landfill, again serving the Tossilo plant, located in Monte-Muradu, in the area north of Macomer.

Although the area is heavily industrialized, the official data published by the health authority and the Environmental Protection Agency have always highlighted parameter values that comply with the regulatory limits, the absence of pollution from the environmental matrices, and excluded an excesses of pathologies in the area of study [39,40,41].

### 2.2. Survey Method

An anonymous questionnaire was built, tested, adjusted, and validated through a pilot study, carried out on a convenience sample of 20 experts in Public Health (data not published). The internal consistency was assessed with Cronbach’s alpha test.

The questionnaire consisted of 14 close-ended questions divided into two areas of investigation: 6 personal data questions; 8 questions related to health concerns and risk perception. To complete the questionnaire it was required to answer each question. Only one question (item no. 13) allowed for more than one answer.

The questionnaire was administered by publishing a Google form URL code in the local and regional newspapers (i.e., “Il Marghine” and “La Nuova Sardegna”), and via social media (i.e., Facebook public profiles of the same newspapers). The questionnaire was to be completed in the period between 1st September 2018 and 31st December 2018. The full questionnaire is shown in Table 2 and Table 3 (Results Section): Table 2 reports 6 questions related to the respondents’ general information; Table 3 shows 8 questions (numbers 7 to 14) related to health concerns and risk perceptions, together with their close-ended answers. Questions and answers are reported in the first and second columns of the tables, respectively.

### 2.3. Statistical Analysis

Data were entered on Excel (Microsoft Office, Microsoft Corporation, Redmond, WA, USA) and analyzed using the STATA software 16 (StatCorp., Austin, TX, USA). Qualitative variables were summarized with absolute and relative (percentage) frequencies.

The differences between mean values for quantitative variables were tested applying the Student t-test, whereas for proportions, Z test was applied. The independence for qualitative variables was tested applying the Χ^2^ test. In order to evaluate the equality of distributions, Kolgomorov Smirnov test for two samples was performed. A *p*-value less than 0.05 was considered statistically significant.

## 3. Results

### 3.1. General Information

With regard to the internal consistency, the questionnaire showed a Cronbach’s alpha reliability test global value of 0.9044, which highlights a very good internal consistency. No missing data were managed.

During the observation period, 651 residents in the study area voluntarily answered the questionnaire. The respondents’ general information related to the first six questions is shown in Table 3.

Of the 651 respondents, 500 were from Macomer, whereas 151 lived in the other municipalities in Marghine. As regards the age and gender of the respondents, the average age was 38.7 years (±13.8), without statistically significant differences between gender, and more than half of the self-selected sample were between 18 and 39 years old. As regards the equality of the distribution by age groups, no differences were observed between genders (combined Kolmogorov-Smirnoff K-S = 0.05; *p* = 0.82). Moreover, 64.4% were female.

As regards marital status, most of the respondents were unmarried, and this percentage was in line with that of the general population (45.3% and 45.0%, respectively). All age groups had at least one respondent.

Over 80% of the sample interviewed had a high school diploma or a university degree, while more than half said they were in employment. Finally, 89.9% of the respondents had resided in the study area for over 10 years.

### 3.2. Descriptive Analysis

The results of the descriptive analysis are shown in Table 4 (questions 7 to 14).

**Figure 3:**
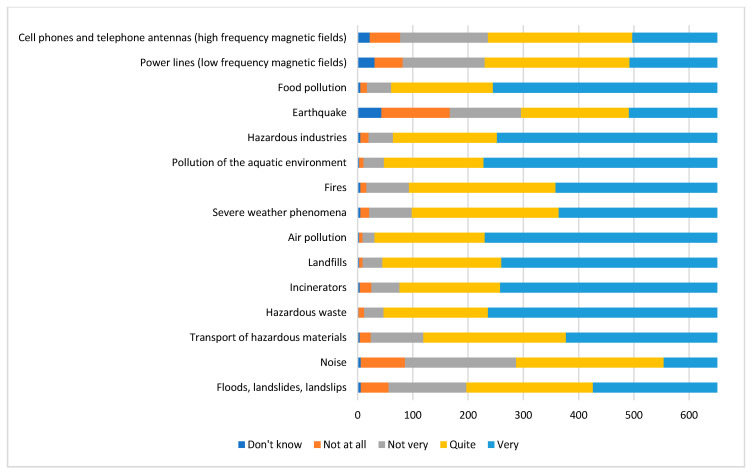
Perception of health problems declared by respondents.

As regards the respondents’ perception in relation to the environmental problems reported in question 7, the results are shown graphically in Figure 3.

In particular, the interviewees showed quite a high level of concern regarding all the environmental problems investigated. As the graphic shows, the events that cause the greatest worry are air pollution, with 95.3%, and hazardous waste, with 92.7% of the respondents stating that they perceived the problem as “very” or “quite” worrying, respectively. These latter two figures (air pollution and hazardous waste) seem to significantly differ between genders, with a concern proportion, from quite to very high, of 91.8% vs. 97.1% and 88.8% vs. 95.0%, for males and females, respectively. Statistically significant differences for the same two figures were also observed among age groups (*p* < 0.001). In particular, the concern seems to grow according to age, ranging from 75.0% in <18 years old to 98.4% in 60–69 years old.

**Figure 4:**
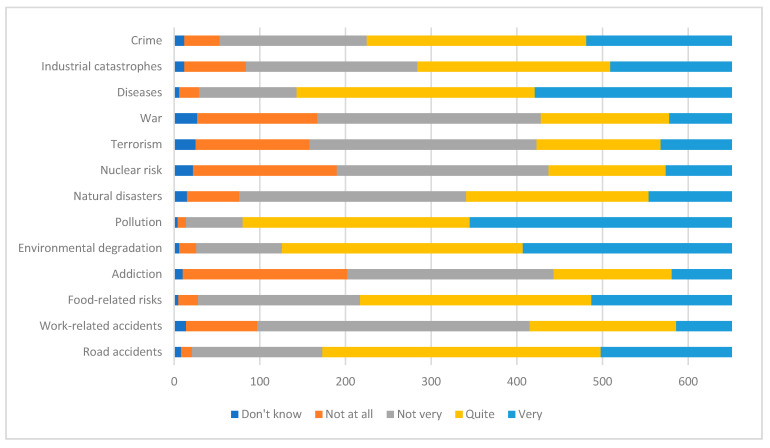
Health-threatening events declared by respondents.

The answers to question 8 are shown in Figure 4.

The events that respondents found less likely are war, terrorism, nuclear risk, and addiction, while diseases were considered to be the most likely event.

**Figure 5:**
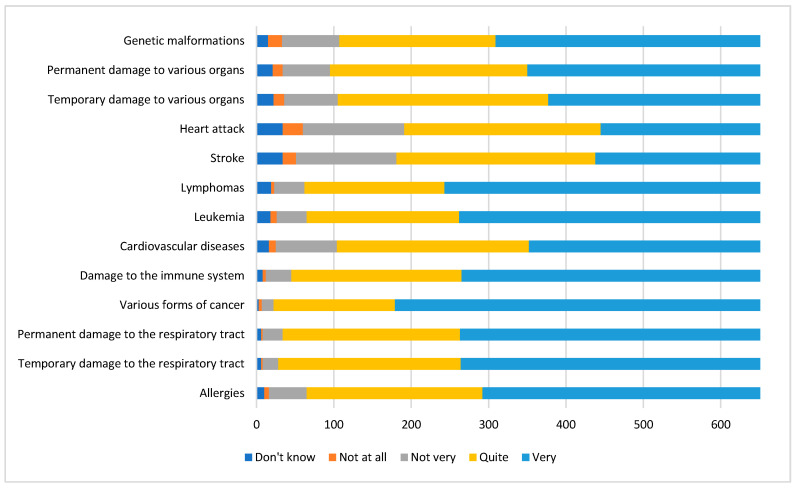
Health concerns declared by respondents.

Figure 5 shows the results of the answers given to question no. 9, concerning worries about one’s own health regarding environmental determinants.

As the figures show, all the health problems investigated in relation to environmental quality aroused concern among the interviewees, in particular tumors and (temporary or permanent) damage to the respiratory tract, without statistically significant differences between gender and age groups.

Finally, the results of the questionnaire show that 93.1% believed there was a cause-effect relationship between environmental quality and health (question no. 10), 60.3% believed that the environmental situation in the area was serious (question no. 11) and 63% believed that citizens do not have an influential role in decisions made by the municipal administration (question no. 12).

### 3.3. Information Sources

**Figure 6:**
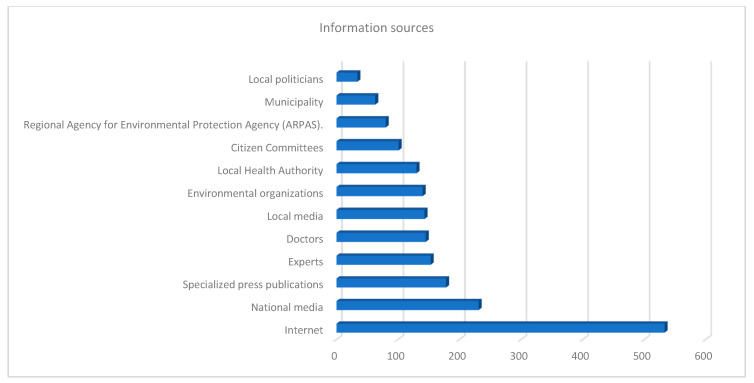
Main sources of information.

Question number 13 revealed the respondents’ main sources of information (more than one answer was allowed) and the answers are shown in Figure 6.

As can be seen from the graph, the Internet was found to be the most widely used source of information, as opposed to consulting political decision-makers, Municipalities, and the Regional Agency for Environmental Protection Agency (ARPAS).

Finally, question number 14 highlighted the willingness of over 40% of the interviewees to relocate away from their place of residence.

## 4. Discussion

The survey enabled an evaluation of environmental risk perception in a self-selected sample of a population living near industrial plants with a high emotional impact. The strengths and weaknesses presented in the study are discussed in this section.

During the observation period, 651 people responded to the survey, with female respondents more numerous. This figure is attributable to the fact that in the Region, and in the area subject to observation, the female population outnumbers the male. Furthermore, as is known, the female population is more sensitive than the male to environmental issues. For this reason, the greater frequency of female respondents is in line with what has been reported in other similar surveys [42].

Of the 651 respondents, 500 claimed to reside in Macomer. This, on the one hand, is attributable to the fact that the Municipality counted almost a half of the population of the entire area observed at the time of the investigation; on the other, the fact that the main industrial plants in the area (i.e., waste-to-energy plants, landfills, and purifiers) were in close proximity to the town center could explain the citizens’ greater sensitivity toward this investigation.

As far as the self-selected sample’s general information is concerned, over half of the respondents were aged between 18 and 39 years, with an average age of 38.7 years. In particular, with regard to the population of Sardinia (average age of 45.9 years), and of Marghine (average age of 47.9 years), the respondents were younger. Nevertheless, approximately 90% of those surveyed said they had lived in the area for more than 10 years. Although, on the one hand, the way the questionnaire was administered may have favored a response by people more inclined toward the use of IT tools, on the other hand, all age groups are represented in the survey.

Another interesting result, in line with what has been described on the international scene, was the link between educational qualifications and perception of environmental risks. Looking at the sample of respondents to the survey, over 80% of them had a high school diploma or university degree. As previously stated by Carducci et al. and by Ozdemir et al. [43,44], subjects with a higher level of education perceive environmental risks to be higher.

In general, there was a clear concern among respondents toward environmental determinants, both in relation to the perception of risks and possible effects on health, with 93.1% of respondents claiming the existence of a clear cause-effect relationship between the state of the environment and health status. Consequently, all of the environmental problems investigated worried the majority of respondents.

In particular, percentages equal to or even greater than 90% were observed in relation to the presence of hazardous industries, particularly landfills and incinerators/waste-to-energy plants. As recently observed by other authors [11,45], these structures play an important role in environmental risk perception in populations exposed to them, and could explain the consequent high level of concern regarding food and aquatic environment pollution, hazardous material transportation, noise, and air pollution, and hazardous waste. As can be expected, these latter are closely related to the presence of industrial plants and may explain the citizens’ apprehension about possible industrial catastrophes and long-term damage to health that emerged from this survey. In particular, a very high concern was observed among females and older age groups. These figures confirm the aforementioned statement that females are more sensitive than males to environmental issues. Moreover, it seems interesting to point out the higher concern among older individuals. Considering the younger mean age of our respondents compared to the general population, this aspect could imply that the real concern could be even greater.

Moreover, the self-selected respondents were concerned by severe weather phenomena (44.1%) and fires (45.0%). On the one hand, the catastrophes caused by extreme weather events that have hit Italy [46] and Sardinia [47] in recent years have certainly influenced the current fear of such an event. On the other hand, the concern about fires is not surprising, given that these events are frequent in the Region [48].

It is also interesting to note two peculiar conditions declared by the interviewees. First, although Sardinia is a region with moderate seismic hazard [49], more than half of the respondents (54.6%) said they were worried by earthquakes. This particular fact could be traced back to the seismic events occurring in Central Italy starting from 2016 [50] which were most likely, according to the dynamics of availability bias, responsible for people’s tendency to base their judgments on recent information, forming opinions conditioned by the latest news acquired [51,52].

Second, a factor that caused little concern was terrorism. In this case, contrary to what was described above, a terrorist cell had been uncovered in Macomer shortly before the present investigation [53], proving the gap that can be found in a population between the perception of a risk (outrage) compared to the real danger (hazard) [16,54].

With regard to road or work accidents, however, a mixed feeling of exposure emerged from the investigation. On the one hand, in fact, respondents showed a high perception of the risk inherent in road accidents. In this case, as found in accordance with the investigations proposed by the Italian National Statistics Institute and by Congiu et al. [55,56], this phenomenon not only represents a known public health problem, but is also increasing if we take fragile categories into consideration.

On the other hand, however, the scant perception of the danger inherent in accidents at work could be attributed to the fact that almost 50% of the volunteer participants were unemployed, students, housewives, or occasional workers.

As for the perception of the environmental quality of the area of residence, although the official analyses carried out by the Regional Environmental Protection Agency of Sardinia excluded the presence of pollutants in the environmental matrices at the time of investigation [39], and excessive numbers of disease cases have not been reported by health authorities [41], the concern of respondents is tangible. Nevertheless, a possible determinant of the outrage could be identified in a controversy that arose in the territory understudy at the end of 2015. In fact, at that time the conversion of the incinerator into a waste-to-energy plant had started and an environmentalist association was in opposition to this transformation. This association claimed that there was an excess of pathologies and specific mortality caused by tumors in that territory. The data on which this position were based derived in part from some incorrect health statistics that had been published in that period [57], in part from an incorrect reading and interpretation of available epidemiological data. Not even the subsequent correction and publication of correct data [41,58] was able to quell the controversy and judicial investigations were also initiated which had no effect and the waste-to-energy plant was set up.

Even some time later, the sense of bewilderment so clearly raised by the numerous newspaper articles that appeared in the local media for several months still remains strong. This fact could explain why the concern for an excess of tumors was high in both sexes, without significant differences.

As Jonathan Swift said, “Falsehood flies and truth comes limping after; so that when men come to be undeceived it is too late; the jest is over and the tale has had its effect” [59].

This situation is aggravated by the fact that most respondents (over 60%) believed that citizens do not play an influential role in the decisions made by the municipal administration. In fact, as highlighted by Peter Sandman himself [27] with the aforementioned outrage theory, the perception of a risk increases when the situation that generates it is independent of one’s will and is attributable to third parties.

Furthermore, over 40% of respondents declared the will to relocate. This figure appears to be in contrast with the Sardinian people’s well-known sense of belonging and attachment to their land, as well as antithetical to the fact that almost 90% of the replies had been residing in the area for more than 10 years. Nevertheless, since the question referring to the will to relocate in relation only to the perceived environmental risks is not explicitly asked, there could be social, economic, and cultural dynamics behind this desire to move away.

## 5. Conclusions

The present study has evaluated the role of environmental risk perception among a self-selected sample of citizens living in an area where industrial plants with high perceptual and emotional impact are situated. In particular, as other studies had previously underlined, the study shows that the perceived risks are not always in line with the real ones, if we think of how, for example, the respondents answered regarding fear of earthquakes, highly unlikely events in the territory under observation. Thus, it is imperative to articulate interventions that are aimed at offering the population objective tools to enable them to interpret the risks themselves. In this regard, a fundamental role is played by adequate communication between the competent bodies and political decision-makers and the population. Moreover, the study also revealed how the process of participation in decision-making is one of the determining aspects that influences a person’s environmental risk perception, and promoting citizens’ involvement in decisions can strengthen their sense of belonging, attachment to the territory, and empowerment. In fact, any action on the territory and even more so its protection (and consequently the perception of the risk linked to the action) that does not stem from an involvement of the local community, is in vain, as it is not legitimized by the context [60].

Starting from the results of the survey, although the study is descriptive in nature and, therefore, requires further investigation in order to better understand the dynamics underlying the high outrage found, some practical actions could be implemented. These should not only concern informing and educating citizens, but should also be addressed to Health Authorities and Institutions (Municipalities). In particular, the results of this survey could be very useful for the launch of projects in the area that see the active participation of citizens in decision-making. For this reason, it will be necessary to bring into play multiple professional skills, not only public health professionals and sociologists, but also designers, planners, and urban planners.

In addition, an important role is played by journalists, who are responsible for informing citizens. As known, the mass media are often responsible for riding the wave of news stories that attract the attention of readers as they are fueled by an emotional component [15]. For this reason, it would be appropriate to implement a training project that also involves this category of professionals.

Finally, a reflection in light of the pandemic that the world is currently experiencing opens up interesting prospects for this study. Indeed, it is worth questioning whether the desire to leave the territory studied in this paper is not quenched precisely because low population density is perceived as a less effective medium for the spread of viruses such as SARS-CoV-2, and as such the condition of “urbanity” is not such a great benefit now, nor in the future [61,62].

## Figures and Tables

**Table 1:** Distribution of the Sardinian population (2018) by age, marital status, and gender [32].

Age	Unmarried	Married	Widowed	Divorced	Male (%)	Female (%)	Total (%)
0–4	54,772	0	0	0	28,367 (51.8)	26,405 (48.2)	54,772 (3.3)
5–9	65,637	0	0	0	33,931 (51.7)	31,706 (48.3)	65,637 (4.0)
10–14	67,981	0	0	0	35,207 (51.8)	32,774 (48.2)	67,981 (4.1)
15–19	70,818	23	0	1	37,048 (52.3)	33,794 (47.7)	70,842 (4.3)
20–24	74,829	865	2	13	40,194 (53.1)	35,515 (46.9)	75,709 (4.6)
25–29	79,339	7225	12	140	45,031 (51.9)	41,685 (48.1)	86,716 (5.3)
30–34	68,503	23,785	60	582	47,610 (51.2)	45,320 (48.8)	92,930 (5.6)
35–39	58,029	47,109	214	1808	54,563 (50.9)	52,597 (49.1)	107,160 (6.5)
40–44	52,167	72,656	643	3946	65,895 (50.9)	63,517 (49.1)	129,412 (7.9)
45–49	39,820	87,582	1371	6015	67,326 (49.9)	67,462 (50.1)	134,788 (8.2)
50–54	29,837	98,775	2812	7237	68,656 (49.5)	70,005 (50.5)	138,661 (8.4)
55–59	20,420	94,499	4737	6403	61,737 (49.0)	64,322 (51.0)	126,059 (7.6)
60–64	15,220	88,065	7444	4931	56,276 (48.7)	59,384 (51.3)	115,660 (7.0)
65–69	11,930	79,393	11,002	3535	51,154 (48.3)	54,706 (51.7)	105,860 (6.4)
70–74	9405	62,695	14,831	2216	41,979 (47.1)	47,168 (52.9)	89,147 (5.4)
75–79	8344	46,709	20,615	1325	34,116 (44.3)	42,877 (55.7)	76,993 (4.7)
80–84	6552	26,386	21,631	638	22,584 (40.9)	32,623 (59.1)	55,207 (3.3)
85–89	4521	11,885	18,838	248	12,843 (36.2)	22,649 (63.8)	35,492 (2.2)
90–94	2025	3172	9856	59	4524 (29.9)	10,588 (70.1)	15,112 (0.9)
95–99	464	452	2682	16	938 (26.0)	2676 (74.0)	3614 (0.2)
100+	65	30	328	1	93 (21.9)	331 (78.1)	424 (0.0)
Total	740,678	751,306	117,078	39,114	810,072 (49.1)	838,104 (50.9)	1,648,176 (100.0)

**Table 2:** Total inhabitants in Marghine (total) and in the individual municipalities as of 2018 [34].

Municipalities	No. of Inhabitants	% Females	Average Age (Years)
Birori	529	49.3	47.9
Bolotana	2579	53.4	50.4
Borore	2079	51.7	47.3
Bortigali	1335	53.2	51.2
Dualchi	613	51.2	51.9
Lei	499	50.3	51.0
Macomer	10,019	51.4	46.7
Noragugume	309	50.8	48.8
Silanus	2109	49.9	46.5
Sindia	1701	52.1	48.4
Total	21,772	52.3	47.9

**Table 3:** Respondents general information.

General Information	Answers	Number	%
1. Gender	Male	232/651	35.6
2. Age (years)	<18	4/651	0.6
	18–29	227/651	34.9
	30–39	151/651	23.2
	40–49	108/651	16.6
	50–59	91/651	14.0
	60–69	62/651	9.5
	70–79	7/651	1.1
	>80	1/651	0.2
3. Educational Attainment	Elementary school	2/651	0.3
	Middle school	93/651	14.3
	High school Diploma	326/651	50.1
	University Degree	230/651	35.3
4. Occupation	Unemployed	71/651	10.9
	First-time job-seeker	15/651	2.3
	Homemaker	52/651	8.0
	Student	98/651	15.1
	National service	2/651	0.3
	Occasional/seasonal worker	41/651	6.3
	Retired	36/651	5.5
	Fixed-term employee	102/651	15.7
	Permanent employee	234/651	35.9
5. Family Unit Composition	Single/Unmarried	295/651	45.3
	Couple without children	85/651	13.1
	Couple with children	238/651	36.6
	Lone parent	19/651	2.9
	Widower/Widow	14/651	2.2
6. Duration of Residence	At least one year	16/651	2.5
	2–5 years	26/651	4.0
	5–10 years	24/651	3.7
	More than 10 years	585/651	89.9

**Table 4:** Questionnaire items and sub-items and descriptive analysis.

7. How Concerned Are You by the Following Environmental Problems?			
7.1 Floods, landslides, landslips	Don’t know	6/651	0.9
	Not at all	50/651	7.7
	Not very	141/651	21.7
	Quite	229/651	35.2
	Very	225/651	34.6
7.2 Noise	Don’t know	6/651	0.9
	Not at all	80/651	12.3
	Not very	201/651	30.9
	Quite	267/651	41.0
	Very	97/651	14.9
7.3 Transport of hazardous material	Don’t know	4/651	0.6
	Not at all	20/651	3.1
	Not very	95/651	14.6
	Quite	258/651	39.6
	Very	274/651	42.1
7.4 Hazardous waste	Don’t know	2/651	0.3
	Not at all	10/651	1.5
	Not very	35/651	5.4
	Quite	189/651	29.0
	Very	415/651	63.7
7.5 Incinerators	Don’t know	4/651	0.6
	Not at all	21/651	3.2
	Not very	51/651	7.8
	Quite	182/651	28.0
	Very	393/651	60.4
7.6 Landfills	Don’t know	3/651	0.5
	Not at all	6/651	0.9
	Not very	36/651	5.5
	Quite	215/651	33.0
	Very	391/651	60.1
7.7 Air pollution	Don’t know	3/651	0.5
	Not at all	6/651	0.9
	Not very	22/651	3.4
	Quite	199/651	30.6
	Very	421/651	64.7
7.8 Severe weather phenomena	Don’t know	5/651	0.8
	Not at all	16/651	2.5
	Not very	77/651	11.8
	Quite	266/651	40.9
	Very	287/651	44.1
7.9 Fires	Don’t know	5/651	0.8
	Not at all	11/651	1.7
	Not very	77/651	11.8
	Quite	265/651	40.7
	Very	293/651	45.0
7.10 Pollution of the aquatic environment	Don’t know	3/651	0.5
	Not at all	8/651	1.2
	Not very	37/651	5.7
	Quite	180/651	27.6
	Very	423/651	65.0
7.11 Hazardous industries	Don’t know	5/651	0.8
	Not at all	15/651	2.3
	Not very	44/651	6.8
	Quite	188/651	28.9
	Very	399/651	61.3
7.12 Earthquake	Don’t know	43/651	6.6
	Not at all	124/651	19.0
	Not very	129/651	19.8
	Quite	195/651	30.0
	Very	160/651	24.6
7.13 Food pollution	Don’t know	5/651	0.8
	Not at all	12/651	1.8
	Not very	44/651	6.8
	Quite	184/651	28.3
	Very	406/651	62.4
7.14 Power lines (low frequency magnetic fields)	Don’t know	31/651	4.8
	Not at all	51/651	7.8
	Not very	148/651	22.7
	Quite	262/651	40.2
	Very	159/651	24.4
7.15 Cell phones and telephone antennas (high frequency magnetic fields)	Don’t know	22/651	3.4
	Not at all	55/651	8.4
	Not very	159/651	24.4
	Quite	261/651	40.1
	Very	154/651	23.7
8. To what extent do you feel exposed to each of these events?			
8.1 Road accidents	Don’t know	8/651	1.2
	Not at all	13/651	2.0
	Not very	152/651	23.3
	Quite	325/651	49.9
	Very	153/651	23.5
8.2 Work-related accidents	Don’t know	14/651	2.2
	Not at all	83/651	12.7
	Not very	318/651	48.8
	Quite	171/651	26.3
	Very	65/651	10.0
8.3 Food-related risks	Don’t know	5/651	0.8
	Not at all	23/651	3.5
	Not very	189/651	29.0
	Quite	270/651	41.5
	Very	164/651	25.2
8.4 Addiction	Don’t know	10/651	1.5
	Not at all	192/651	29.5
	Not very	241/651	37.0
	Quite	138/651	21.2
	Very	70/651	10.8
8.5 Environmental degradation	Don’t know	6/651	0.9
	Not at all	20/651	3.1
	Not very	100/651	15.4
	Quite	281/651	43.2
	Very	244/651	37.5
8.6 Pollution	Don’t know	4/651	0.6
	Not at all	10/651	1.5
	Not very	66/651	10.1
	Quite	265/651	40.7
	Very	306/651	47.0
8.7 Natural disasters	Don’t know	15/651	2.3
	Not at all	61/651	9.4
	Not very	265/651	40.7
	Quite	213/651	32.7
	Very	97/651	14.9
8.8 Nuclear risk	Don’t know	22/651	3.4
	Not at all	168/651	25.8
	Not very	247/651	37.9
	Quite	137/651	21.0
	Very	77/651	11.8
8.9 Terrorism	Don’t know	25/651	3.8
	Not at all	133/651	20.4
	Not very	265/651	40.7
	Quite	145/651	22.3
	Very	83/651	12.7
8.10 War	Don’t know	27/651	4.1
	Not at all	140/651	21.5
	Not very	261/651	40.1
	Quite	150/651	23.0
	Very	73/651	11.2
8.11 Diseases	Don’t know	6/651	0.9
	Not at all	23/651	3.5
	Not very	114/651	17.5
	Quite	278/651	42.7
	Very	230/651	35.3
8.12 Industrial catastrophes	Don’t know	12/651	1.8
	Not at all	72/651	11.1
	Not very	200/651	30.7
	Quite	225/651	34.6
	Very	142/651	21.8
8.13 Crime	Don’t know	12/651	1.8
	Not at all	41/651	6.3
	Not very	172/651	26.4
	Quite	256/651	39.3
	Very	170/651	26.1
9. In your opinion, how likely it is for those who live near a polluted area to contract one of the following?			
9.1 Allergies	Don’t know	10/651	1.5
	Not at all	6/651	0.9
	Not very	49/651	7.5
	Quite	227/651	34.9
	Very	359/651	55.1
9.2 Temporary damage to the respiratory tract	Don’t know	6/651	0.9
	Not at all	2/651	0.3
	Not very	20/651	3.1
	Quite	236/651	36.3
	Very	387/651	59.4
9.3 Permanent damage to the respiratory tract	Don’t know	6/651	0.9
	Not at all	2/651	0.3
	Not very	26/651	4.0
	Quite	229/651	35.2
	Very	388/651	59.6
9.4 Various forms of cancer	Don’t know	3/651	0.5
	Not at all	4/651	0.6
	Not very	15/651	2.3
	Quite	157/651	24.1
	Very	472/651	72.5
9.5 Damage to the immune system	Don’t know	8/651	1.2
	Not at all	4/651	0.6
	Not very	33/651	5.1
	Quite	220/651	33.8
	Very	386/651	59.3
9.6 Cardiovascular diseases	Don’t know	16/651	2.5
	Not at all	9/651	1.4
	Not very	79/651	12.1
	Quite	248/651	38.1
	Very	299/651	45.9
9.7 Leukemia	Don’t know	18/651	2.8
	Not at all	8/651	1.2
	Not very	39/651	6.0
	Quite	197/651	30.3
	Very	389/651	59.8
9.8 Lymphomas	Don’t know	19/651	2.9
	Not at all	4/651	0.6
	Not very	39/651	6.0
	Quite	181/651	27.8
	Very	408/651	62.7
9.9 Stroke	Don’t know	34/651	5.2
	Not at all	17/651	2.6
	Not very	130/651	20.0
	Quite	257/651	39.5
	Very	213/651	32.7
9.10 Heart attack	Don’t know	34/651	5.2
	Not at all	26/651	4.0
	Not very	131/651	20.1
	Quite	254/651	39.0
	Very	206/651	31.6
9.11 Temporary damage to various organs	Don’t know	22/651	3.4
	Not at all	14/651	2.2
	Not very	69/651	10.6
	Quite	272/651	41.8
	Very	274/651	42.1
9.12 Permanent damage to various organs	Don’t know	21/651	3.2
	Not at all	13/651	2.0
	Not very	61/651	9.4
	Quite	255/651	39.2
	Very	301/651	46.2
9.13 Genetic malformations	Don’t know	15/651	2.3
	Not at all	18/651	2.8
	Not very	74/651	11.4
	Quite	202/651	31.0
	Very	342/651	52.5
10. Do you believe there is a cause-effect relationship between the state of the environment and health?	No	12/651	1.8
	Yes	606/651	93.1
	I am not informed	33/651	5.1
11. How would you define the environmental situation in the municipality where you live?	Excellent	30/651	4.6
	Acceptable	228/651	35.0
	Serious but solvable	366/651	56.2
	Serious and unsolvable	27/651	4.1
12. In your opinion, does the citizen have an influential role in the decisions made by the municipal administration?	Don’t know	20/651	3.1
	Not at all	168/651	25.8
	Not very	244/651	37.5
	Quite	138/651	21.2
	Very	81/651	12.4
13. By what means do you usually keep up-to-date about the risks to which you are exposed? (more than one answer is allowed)	Internet	533/651	81.9
	Environmental organizations	140/651	21.5
	National media	231/651	35.5
	Municipality	63/651	9.7
	Experts	153/651	23.5
	Local Health Authority	130/651	20.0
	Specialized press publications	178/651	27.3
	Citizen Committees	101/651	15.5
	Local media	143/651	22.0
	Doctors	145/651	22.3
	Local politicians	34/651	5.2
	Environmental Protection Agency	80/651	12.3
14. Would you leave the area in which you live?	Yes	263/651	40.4
	No	388/651	59.6
